# Racial and Ethnic Disparities in Dietary Intake and Quality Among United States Veterans

**DOI:** 10.1016/j.cdnut.2024.104461

**Published:** 2024-09-29

**Authors:** Xuan-Mai T Nguyen, Yanping Li, Stacey B Whitbourne, Luc Djousse, Dong D Wang, Kerry Ivey, Walter C Willett, John Michael Gaziano, Kelly Cho, Frank B Hu, VA Million Veteran Program

**Affiliations:** 1 Million Veteran Program Boston Coordinating Center, VA Boston Healthcare System, Boston, MA, United States; 2 Department of Internal Medicine, UCLA David Geffen School of Medicine, Los Angeles, CA, United States; 3 Department of Nutrition, Harvard T. H. Chan School of Public Health, Boston, MA, United States; 4 Division of Aging, Brigham and Women’s Hospital, Boston, MA, United States; 5 Department of Medicine, Harvard Medical School, Boston, MA, United States; 6 The Channing Division for Network Medicine, Department of Medicine, Brigham and Women’s Hospital and Harvard Medical School, Boston, MA, United States; 7 Broad Institute of MIT and Harvard, Cambridge, MA, United States

**Keywords:** veterans, racial disparities, dietary intake, dietary pattern, DASH

## Abstract

**Background:**

Dietary quality plays an important role in disease development and prognosis, and diet is also a key contributor to disparities in many chronic diseases and health conditions.

**Objectives:**

This study aimed to assess racial and ethnic disparities experienced by veterans; we examined food intake and dietary quality across different racial and ethnic groups of United States veterans.

**Methods:**

The study included 420,730 males and females aged 19–107 y (91.2% males) enrolled in the Veterans Affairs Million Veteran Program with plausible dietary intake measured by food frequency questionnaire. Dietary quality was evaluated with dietary approaches to stop hypertension (DASH) score. Dietary intakes of various race and ethnicity groups were standardized to the age distribution of non-Hispanic White participants, separately for males and females. Differences across race and ethnicity groups were compared using general linear regression models after adjustment for socioeconomic and lifestyle factors as well as military service.

**Results:**

Compared to non-Hispanic White males, non-Hispanic Black males had a relatively lower DASH score, higher red and processed meats, higher sugar-sweetened beverages (SSBs), and lower low-fat dairy intakes. Non-Hispanic Asian males had a relatively higher DASH score as compared to non-Hispanic White males with relatively higher intakes of fruits and vegetables and relatively lower intakes of sodium, red meat and SSBs. Age-standardized DASH scores of Hispanic males and “Other” race/ethnicity groups were not statistically different from non-Hispanic White males. Similar race and ethnicity dietary patterns were found in females, although not all reached a statistically significant level.

**Conclusions:**

A modest difference in overall dietary quality (i.e., DASH score) was observed, but unique differences in food preferences across the different racial/ethnic groups were identified. Findings from our study may provide insight for the potential development of specific interventions to help address nutritional disparities experienced among veterans.

## Introduction

Racial and ethnic disparities have been reported in a variety of populations [1,2] including that of veterans [3,4]. Within the Veterans Affairs (VA) Million Veteran Program (MVP), Black, Hispanic, and males and females of other races or ethnicities are more likely to self-report having “fair” to “poor” health compared to White veterans [4]. Additionally, when compared with non-Hispanic White veterans, Black veterans of both genders reported a higher prevalence of circulatory system diseases, whereas Hispanic male veterans reported a significantly lower prevalence of these conditions [4]. Aligned with observed disparities in disease susceptibility across racial and ethnic groups, Black veterans were less likely to have well-controlled cholesterol, blood pressure, and glucose levels than White veterans with access to the same medical facilities. This is despite improvements in standardized measures of care, suggesting that racial and ethnic health disparities are not completely accounted for by access and equality of care [3].

To better understand the burden of various health conditions and underlying risk factors, studies within the VA population recently compared racial and ethnic differences in self-reported health characteristics. It was reported that racial and ethnic disparities exist across several lifestyle factors [3]. For example, rates of tobacco usage were higher among Black males, but lower for other minority groups compared to Whites. Additionally, minority groups were less likely to consume alcohol but reported having lower physical fitness than Whites [3]. These differences in lifestyle factors across racial and ethnic groups may play a role in the observed disparities in chronic disease burden related to race and ethnicity.

Nutrition is an important risk factor associated with chronic disease. It is well-known that dietary quality plays an important role in disease development and prognosis, and diet is also a key contributor to disparities in many chronic diseases and health conditions [1,5]. For example, the Dietary Approaches to Stop Hypertension (DASH) [6] score is a comprehensive summary of dietary factors associated with blood pressure control that emphasizes a higher intake of fruits, vegetables, whole grains, nuts and beans, and low-fat dairy products in addition to a lower intake of sodium, red meat, and sugar-sweetened beverages (SSBs). Higher DASH score has been consistently associated with improved outcomes for not only hypertension but also other chronic diseases and premature death across different racial and ethnic groups [[7], [8], [9]]. In addition, the DASH score is highly correlated with other commonly used diet quality scores such as Alternate Mediterranean Diet Index, Alternate Healthy Eating Index, and Healthy Eating Index-2010 [[7], [8], [9]]. Within the veteran population, dietary quality has not been previously assessed outside of MVP [10]. Additionally, dietary studies among veterans have only focused on eating behaviors and patterns of dietary intake without clear understanding of how diet impacts risk of disease development or how it varies across different racial and ethnic groups [[11], [12], [13], [14]]. The purpose of this research, therefore, was to describe racial and ethnic differences in food intake and dietary quality reflected by the DASH score among males and females in MVP with the goal of understanding how it may impact the prevention and management of chronic diseases.

## Methods

### Study population

MVP is a nationally representative, prospective cohort study of veterans designed to investigate genetic and non-genetic determinants of chronic diseases. Details of MVP study design can be found elsewhere [15,16]. Briefly, beginning in early 2011, MVP began enrolling individuals receiving routine medical care in the United States Department of Veterans Affairs Healthcare System and collecting participant data from self-reported surveys and electronic health records. All participants signed informed consent, and the Veterans Affairs Central Institutional Review Board approved the study protocol (protocol: MVP000, date of approval: 2010) [15,16]. The study was conducted according to the guidelines of the Declaration of Helsinki.

As of August 2023, 963,753 veterans were enrolled with 447,259 participants completing the MVP Lifestyle Survey that includes a semiquantitative food frequency questionnaire (sFFQ) with 61 food items, in addition to questions about added sugar to diet, fried food frequency, and 21 nutritional supplements over the 12 months preceding questionnaire administration [17]. For this analysis, we excluded participants who did not provide dietary information, reported implausible dietary data (i.e., total energy intake: <400 or ≥4000 kcal/d for females; <450 or ≥4500 kcal/d for males) or returned their sFFQ with ≥1 page of blank responses. After these exclusions, the study population consisted of 420,730 participants of which 95% were males with a mean age of 66.7 ± 11.6 y and 5% were females with a mean age of 54.7 ± 12.9 y. The demographics of this final study population were comparable to the 26,529 participants who were excluded (94% males aged 68.6 ± 11.8 y and 6% females aged 56.4 ± 13.5 y).

### Data collections and assessments

Race was assessed with the question “What is your race?”, and responses included the following: White, Black/African American, American Indian/Alaska Native, Chinese, Japanese, Asian Indian, Other Asian, Filipino, Pacific Islander, and other. Ethnicity was assessed with the question “Are you Spanish, Hispanic, or Latino?” Responses included “No, not Spanish, Hispanic, Latino,” “Yes, Mexican, Mexican American, Chicano,” “Yes, Puerto Rican,” “Yes, Cuban,” and “Yes, other Spanish, Hispanic, Latino.” For the main analysis, race and ethnicity were combined into the following categories: White (non-Hispanic), Black (non-Hispanic), Asian (non-Hispanic), Hispanic, and "Other" race/ethnicity (including non-responders to race and/or non-responders to ethnicity). In a secondary analysis, we compared the differences across different Asian origin groups: Chinese, Japanese, Asian Indian, Filipino, and Other Asians.

Participants self-reported their dietary intake through sFFQ, which has demonstrated reasonable validity in assessing intakes of individual foods and nutrients in other cohorts [[18], [19], [20]]. Participants were asked how often, on average, they consumed a standard portion of each food in the past year. Frequencies and portions of each individual food item were converted to average daily intake for each participant. For blank responses to individual food items (∼1%–5% for each food item), we coded it as zero assuming non-consumption or non-response. We conducted a sensitivity analysis among participants without any blank responses for food items included in the DASH score calculation. Sodium intake and total energy intake were calculated by multiplying the frequency of consumption for each food item by its energy and sodium content from the Harvard University Food Composition Database [21] and summing across all foods. Sodium intake was adjusted for total energy intake using a residual method [22].

The overall dietary quality was evaluated with DASH score, based on consumption of 8 items: fruits, vegetables, nuts and legumes, low-fat dairy products, whole grains, SSBs, red and processed meats, and energy-adjusted sodium intake [6]. For each component, we classified participants into quintiles according to intake ranking (ranging from 1 to 5; 5 being the best score corresponding to higher intake of fruits, vegetables, nuts and legumes, low-fat dairy products, and whole grains; 5 being the best score corresponding to lower intakes of sodium, SSBs, and red and processed meats). Total DASH score ranged from 8 to 40 points. Information on covariates, including age, BMI (kg/m^2^), socioeconomic status, current marriage status, education level, physical activity, and branch of services was self-reported.

### Statistical analysis

Direct standardization was used to standardize the DASH score and dietary intakes of food and sodium to the age distribution of non-Hispanic White participants [23,24]. Standardization was performed separately for males and females. Age groups were stratified into 10-y increments (e.g., 30–39 y) except for the youngest (18–29 y) and the oldest (80 y or older for males and 70 y or older for females) age groups. Age-adjusted characteristics for non-Hispanic Black males, Hispanic males, and males in the "Other" race/ethnicity category were compared with those for non-Hispanic White males, separately, using the Cochran–Mantel–Haenszel test for frequencies and a linear regression model for means. Age-adjusted means and frequencies among females were compared similarly.

DASH score and its individual components for non-Hispanic Black males, non-Hispanic Asian males, Hispanic males, and males in the "Other" race/ethnicity category were compared with those for non-Hispanic White males, separately, using a generalized linear model after adjusting for age [<30, 30–39, 40–49, 50–59, 60–69, ≥70 (females), 70–79, and ≥80 (males) y]; education level (≥some college: yes, no, or missing); marital status (married or cohabitating with partner: yes, no, or missing); annual household income (<$30,000, $30,000–$59,999, ≥$60,000, or missing); physical activity level (≥7.5 METs-hours/wk: yes/no); BMI (<25, 25–29, ≥30); and categories for branch of service (Army, Navy, Air Force, Marine Corps, and other or missing). Gender differences between males and females within each racial and ethnic group were analyzed using generalized linear models, adjusting for the aforementioned covariables. *P* values for comparing food/nutrient intake between specific racial/ethnic groups and non-Hispanic Whites were calculated using the Wald test within multivariate-adjusted generalized linear models. Bonferroni correction was used to account for multiple comparisons. Applying a desired α level of 0.05 with 72 comparisons, a corrected α level of 0.0007 was used. These models were analyzed separately for males and females. Analyses were performed using SAS Enterprise Guide 8.2.

## Results

Among 420,730 participants, 383,617 (91.2%) were males with a mean age of 66.7 y and 37,113 (8.8%) were females with a mean age of 54.7 y (Table 1). Among males, 81.2% were non-Hispanic White, 9.3% were non-Hispanic Black, 0.9% were non-Hispanic Asian, 6.1% were Hispanic, and 2.5% were "Other" race/ethnicity. Among females, 70.3% were non-Hispanic White, 17.1% were non-Hispanic Black, 1.3% were non-Hispanic Asian, 8.0% were Hispanic, and 3.4% were "Other" race/ethnicity. Overall, females were younger, were proportionally less likely to be currently married, and possessed a proportionally higher level of education at or above college in comparison to males (Table 1). Regardless of gender, White participants were older, and Asian participants were younger than their respective counterparts in the other racial and ethnic groups in MVP (Table 1). Additionally, Black males were more likely to be current smokers than their counterparts in different racial and ethnic groups. This was not observed among Black females (Table 1).

**TABLE 1:** Baseline characteristics by race and ethnicity.

	Non-Hispanic			Hispanic (26,498)	Other^1^ (10,824)	All (420,730)
	White (337,489)	Black (41,987)	Asian (3932)			
**Males (*****n*****)**	311,418	35,658	3453	23,521	9567	383,617
Proportion (%)	81.2	9.3	0.9	6.1	2.5	
Age (y), mean (SD)	67.7 (11.3)	62.3 (10.4)	60.1 (15.8)	61.9 (13.1)	64.4 (12.7)	66.7 (11.6)
Education, ≥some college (%)	76.7	72.9	89.4	76.5	77.9	76.5
Currently married (%)	67.0	50.6	67.1	61.2	61.0	65.1
Family annual income (%)						
<30k	29.0	40.1	19.1	32.4	34.0	30.1
30k–60k	35.3	32.0	30.4	34.8	34.6	34.9
>60k	35.8	27.9	50.5	32.9	31.4	35.0
Physical activity ≥7.5 MET-h/wk (%)	23.3	17.1	30.8	23.8	23.3	22.9
BMI category (%)						
<25	18.9	18.5	29.4	14.8	16.6	18.6
25–29.9	42.3	38.2	49.8	41.8	41.0	41.9
≥30	38.9	43.3	20.8	43.4	42.4	39.5
Smoking category (%)						
Never smoking	27.3	28.3	37.5	34.0	27.4	27.9
Ever smoking	53.9	42.6	49.3	47.2	51.1	52.3
Current smoking	18.8	29.1	13.2	18.8	21.5	19.8
Branch of service (%)						
Army	46.9	58.1	43.5	53.6	49.1	48.3
Navy	24.2	14.0	34.0	17.9	22.5	23.0
Air force	19.2	17.4	14.6	15.3	17.7	18.7
Marine corps	11.0	12.0	7.9	15.3	12.6	11.3
Others	6.9	5.2	7.2	8.3	5.7	6.8
**Females (*****n*****)**	26,071	6329	479	2977	1257	37,113
Proportion (%)	70.2	17.1	1.3	8.0	3.4	
Age (y), mean (SD)	55.8 (13.1)	53.4 (10.6)	47.6 (13.8)	49.2 (13.2)	53.1 (13.0)	54.7 (12.9)
Education, ≥some college (%)	91.8	93.7	96.5	94.7	93.3	92.5
Currently married (%)	41.4	28.2	45.9	38.4	37.3	39.0
Family annual income (%)						
<30k	29.0	31.8	19.0	25.8	34.5	29.2
30k–60k	31.1	34.1	26.8	33.1	30.9	31.7
>60k	39.9	34.1	54.2	41.1	34.6	39.1
Physical activity ≥7.5 MET-h/wk (%)	28.6	22.2	34.4	30.5	28.6	27.7
BMI category (%)						
<25	27.7	16.2	45.9	24.4	20.0	25.4
25–29.9	30.9	32.4	33.2	33.7	36.9	31.6
≥30	41.4	51.4	20.9	42.0	43.1	42.9
Smoking category (%)						
Never smoking	42.3	51.5	61.6	53.7	41.2	45.1
Ever smoking	37.3	29.4	29.2	31.4	38.0	35.4
Current smoking	20.3	19.1	9.2	14.9	20.8	19.6
Branch of service (%)						
Army	43.3	60.0	47.9	49.6	51.9	46.8
Navy	23.8	15.3	25.4	21.9	21.1	22.3
Air force	26.9	20.5	23.7	21.0	20.7	25.2
Marine corps	6.0	4.0	4.0	7.7	5.8	5.8
Others	8.2	6.4	7.3	7.6	5.6	7.7

1 Other includes veterans who identified as any of the following: American Indian/Alaska Native, Native Hawaiian and other Pacific Islander, or those who did not self-report race and/or ethnicity.

**FIGURE 1:**
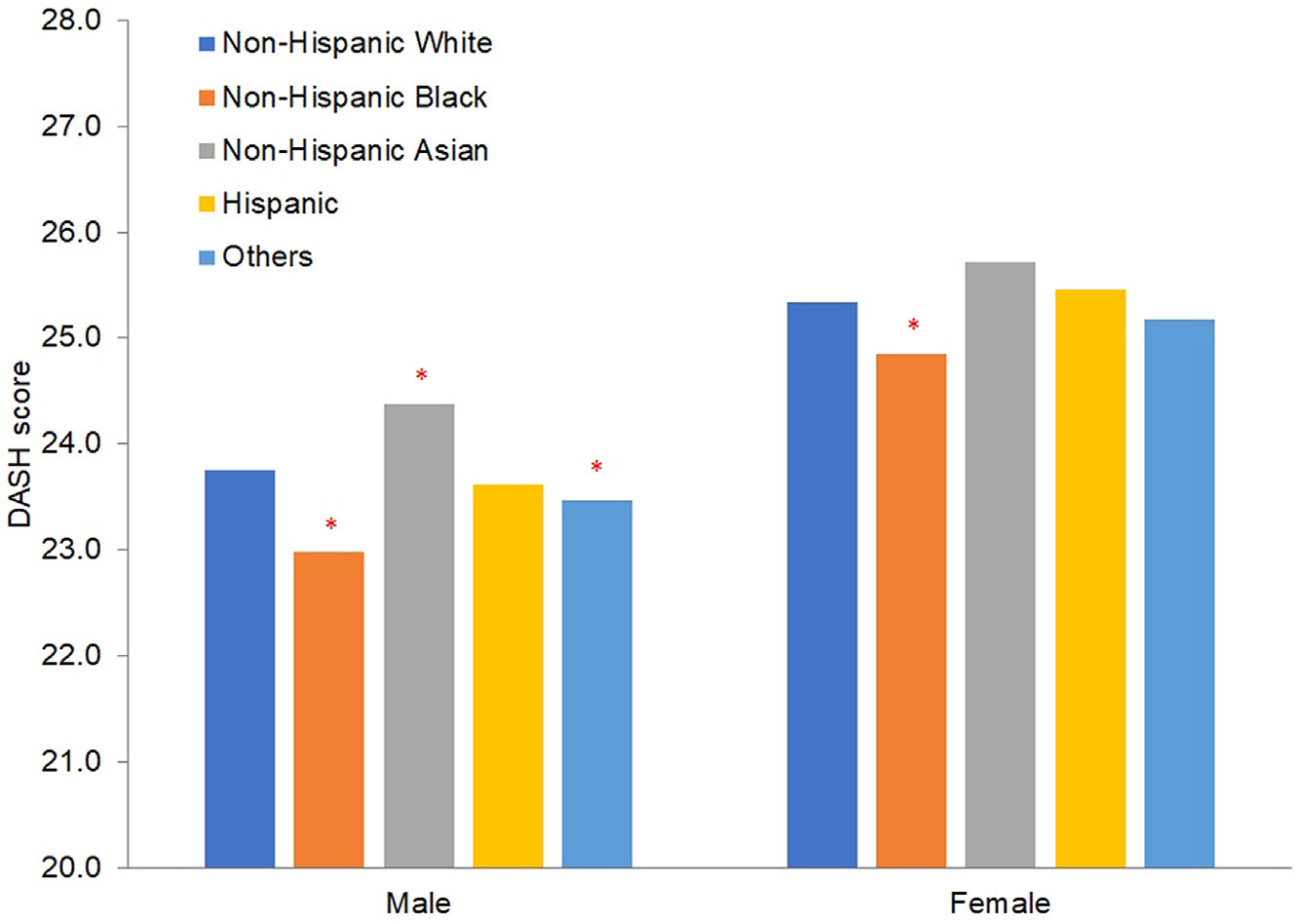
Dietary Approaches to Stop Hypertension (DASH) score for males and females in the VA Million Veteran Program by race and ethnicity. Unadjusted means of non-Hispanic White and age-standardized mean of other race and ethnicity groups, standardized to the age distribution of non-Hispanic White participants. ∗*P* < 0.05 compared with non-Hispanic White after adjustment for age [<30, 30–39, 40–49, 50–59, 60–69, and ≥70 (females), 70–79, and ≥80 (males) y). *P* for gender difference <0.0001 in all race/ethnicity groups.

The mean DASH score among White males was 23.75 (SD: 5.16), 22.99 (SD: 5.21) for Black males, 24.37 (SD: 5.21) for Asian males, 23.62 (SD: 5.20) for Hispanic males, and 23.47 (SD: 5.14) for males of "Other" race/ethnicity (Figure 1). When compared with White males, the age-standardized DASH score was significantly lower among Black males and males of "Other" race/ethnicity. However, the age-standardized DASH score was significantly higher among Asian males as compared to White males (Figure 1), especially among Asian Indians (Supplemental Figure 1). We did not observe a significant difference in age-adjusted DASH scores between Hispanic and White participants (Figure 1). After adjustment of socioeconomic and lifestyle factors in the multivariate analysis, Black males still exhibited a statistically significant lower DASH score compared to White males (Supplemental Table 1).

Among females, the mean DASH score was 25.33 (SD: 5.17) for White participants, 24.86 (SD: 5.32) for Black participants, 25.71 (SD: 5.20) for Asian participants, 25.46 (SD: 5.28) for Hispanic participants, and 25.17 (SD: 5.32) for participants of "Other" race/ethnicity (Figure 1). Females had a significantly higher DASH score than males in all race and ethnicity groups (all *P* < 0.0007). In both age-standardized (Figure 1) and multivariate-adjusted models (Supplemental Table 1), Black females had a significantly lower DASH score than White females. Although Asian females had the highest DASH score compared to other groups, the difference did not reach statistical significance (Supplemental Table 1), which was likely attributable to the small sample size of Asian females in the study. Differences in DASH score across race/ethnicity groups were consistent in the sensitivity analyses that accounted for varying methods of handling missing data in our study (Supplemental Table 2).

**FIGURE 2:**
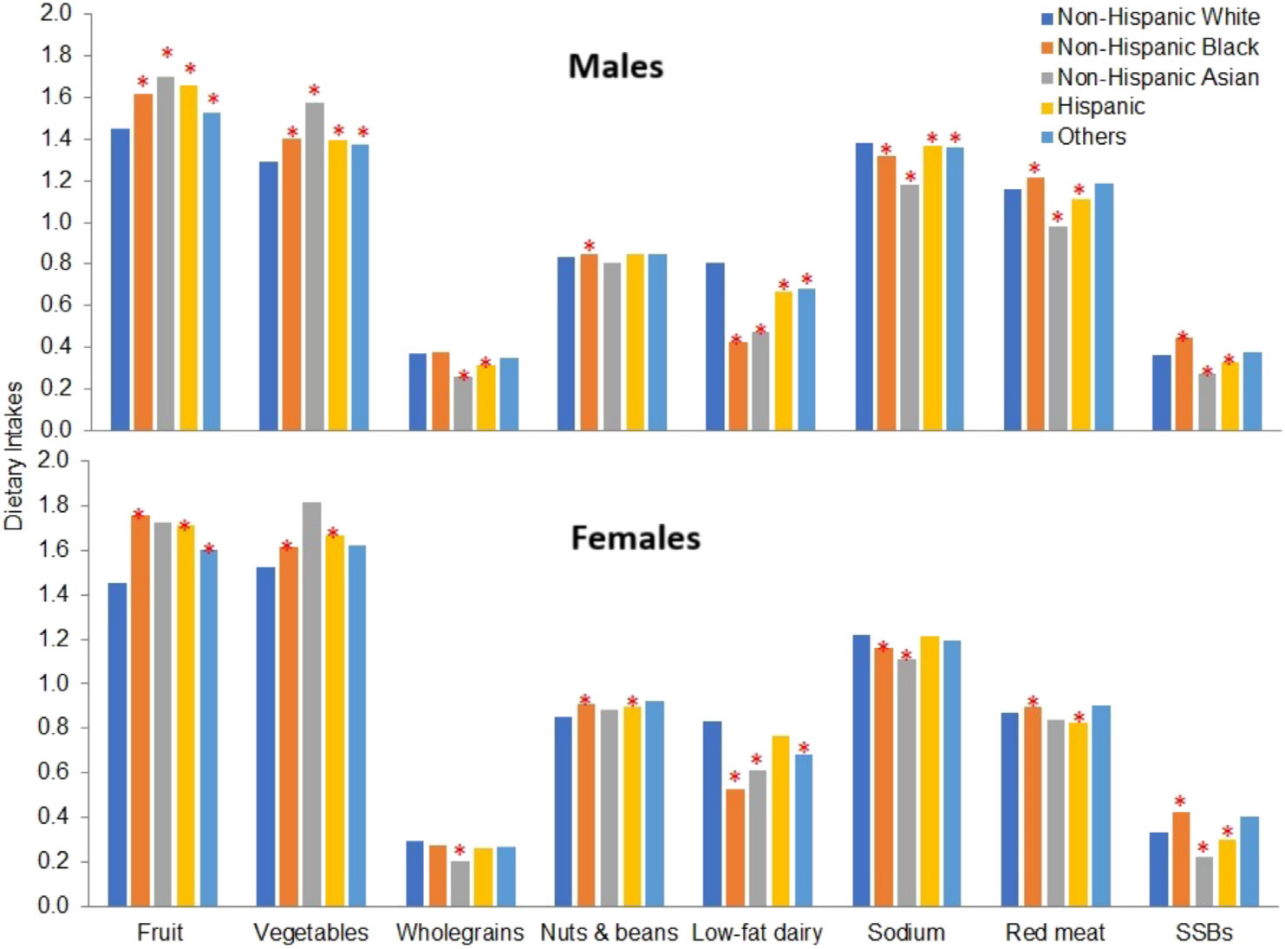
Dietary intakes of males and females in the VA Million Veteran Program by race and ethnicity. X-axis represents servings/day except sodium which is reported in grams per day. Unadjusted means of non-Hispanic White and age-standardized mean of other race and ethnicity groups, standardized to the age distribution of non-Hispanic White participants. ∗*P* < 0.05 compared with non-Hispanic White after adjustment for age [<30, 30–39, 40–49, 50–59, 60–69, ≥70 (females), 70–79, and ≥80 (males) y] and Bonferroni correction for multiple comparisons with a corrected α level of 0.0007.

When food and nutrient intakes were compared across the subgroups, both White males and females had the lowest intake of fruit and vegetables and the highest intake of low-fat dairy compared to "Other" race and ethnicity groups (Figure 2); multiadjustment did not materially change the significance of the differences (Supplemental Table 3). Compared to White males, Black males reported a significantly higher intake of red meat and processed meats, SSBs, fruits, vegetables, nuts and beans, and significantly lower intakes of low-fat dairy and sodium, whereas Asian males had significantly higher intakes of fruits and vegetables but lower whole grain, low-fat dairy, sodium, red meat and processed meats, and SSBs. Hispanic males had significantly higher intakes of fruits and vegetables but significantly lower intakes of sodium, whole grains, low-fat dairy, red meat and processed meats, and SSBs than non-Hispanic White males (Figure 2). Females had a similar race and ethnicity pattern of dietary intake, although some comparisons did not reach statistical significance (Figure 2).

In our secondary analysis examining dietary intake across groups of different Asian origins, males of Asian Indian origin reported higher consumption of fruits, vegetables, whole grains, nuts and beans, and low-fat dairy compared to males of Chinese origin. A similar association was seen among females of Asian Indian origin for whole grains compared to females of Chinese origin. Additionally, the highest intake of fruits and vegetables among females was observed among those of Filipino origin (Supplemental Figure 2).

## Discussion

Based on this large population study of veterans, we observed several racial and ethnic disparities in dietary intake and overall dietary quality, some of which have previously been observed among non-veteran populations [[25], [26], [27]]. The overall difference in dietary quality between White and Black study participants was consistent with findings reported in previous studies with different dietary quality scores [[25], [26], [27], [28]]. The Coronary Artery Risk Development in Young Adults study reported a higher diet score among White participants than among Black study participants [27,28]. Based on the NHANES from 2011 to 2018, the overall dietary quality estimated by Healthy Eating Index 2015 was highest among non-Hispanic Asians, followed by Hispanics, then by non-Hispanic Whites and "Other" race/ethnicity individuals; the lowest overall dietary quality was reported among non-Hispanic Blacks, consistent with race/ethnic disparities of DASH score in this study [26]. In another study based on NHANES 1999–2010 [29], dietary quality estimated by the Alternate Healthy Eating Index 2010 was higher among non-Hispanic Whites than non-Hispanic Blacks in all time periods, which is consistent with our estimations based on DASH score. Socioeconomic status, educational levels, and lifestyle habits are correlated with dietary behaviors [2], and after we further adjusted for these factors in our study, non-Hispanic Black veterans still had significantly lower DASH scores than non-Hispanic Whites.

Previous studies [2,26,30,31] have reported racial and ethnic disparities in food choices among Americans where non-Hispanic Asians, on average, consumed the most fruits, vegetables, and seafood, whereas Hispanics consumed the most meat, non-Hispanic Blacks ate the most poultry, and non-Hispanic Whites consumed the most dairy products, especially cow's milk [31]. Consistent with findings among the general United States population, we found similar racial and ethnic disparities among veterans: non-Hispanic Asians, on average, consumed the most fruits and vegetables, whereas non-Hispanic Blacks consumed the most red meat and processed meat, and non-Hispanic Whites consumed the most low-fat dairy products. International studies indicate that Asians, especially those in East and Central Asia, have the highest sodium consumption in the world [32,33]. However, sodium intake among Asian Americans was not higher than other race and ethnicity groups in both the general United States population of NHANES [30] and veterans in our study. The gender difference in dietary quality among veterans was also consistently observed in the Coronary Artery Risk Development in Young Adults study and NHANES, with females reporting a higher diet score than males regardless of race or time period [26,34].

Clinical trials indicate that a greater adherence to the DASH diet is associated with a larger reduction in blood pressure independent of weight loss [35]. Given that Black veterans were less likely to adhere to the DASH diet than White veterans, this suggests that such disparities may warrant culturally sensitive dietary strategies to improve adherence to the DASH diet [36]. Moreover, knowing that suboptimal diets may increase the risk for non-communicable diseases and total mortality [[37], [38], [39]], understanding variations in dietary quality and food intake among different racial/ethnic groups is essential. This knowledge can guide registered dietitians and other nutrition professionals working with veterans and their families in developing culturally tailored dietary recommendations to promote wellness and prevent disease.

Our study has several strengths, including a large sample size of participants with diverse racial/ethnic backgrounds, comprehensive measurements, careful adjustment for many potential confounders, and dietary intakes based on previously validated sFFQ. Although this study is limited to a single collection of dietary data, it provides a preliminary baseline understanding of the dietary patterns of different racial/ethnic groups in a large veteran population, which has not been previously well-documented. Additional limitations need to be considered when interpreting the results of this research. First, the absolute difference in DASH score across different racial/ethnic groups were small. Although statistically significant, this could be attributed to the study’s large sample size. However, the modest difference between groups in this large population study may still have meaningful significance as the literature has shown that seemingly minor changes in variables, such as BMI, that are measured in large cohort studies have purposeful impact on public health [40,41]. Second, the abbreviated food frequency questionnaire in MVP likely underestimates dietary intake of certain foods. Additionally, some major sources of dietary sodium were missed in the sFFQ, such as salt added at the table and salt used in preparation or cooking [42]. However, the DASH score is based on quintiles derived from the population distribution of sodium intake, so the relative differences observed in dietary quality across race and ethnicity groups would be minimized. Third, measurement errors in dietary assessment are inevitable. However, we were able to reduce the measurement error to a large degree by applying energy adjustments that negated the correlated errors in nutrient and energy intake assessments [22]. Our predominantly male population is another study limitation because it restricted the statistical power of females. Last, but not least, although the sFFQ has demonstrated reasonably well validity in assessing intake of individual foods in other cohorts, validation studies among different race/ethnicity groups are still warranted.

In conclusion, our data suggest that non-Hispanic Black veterans had a relatively lower and non-Hispanic Asian and Hispanic veterans had a higher overall dietary quality as compared with non-Hispanic Whites. Although the absolute difference in DASH scores was modest, the contributing pattern of dietary intake varied across racial/ethnic groups where non-Hispanic Asians consumed the most fruits and vegetables, on average, and non-Hispanic Blacks consumed the most red and processed meats, while non-Hispanic Whites consumed the most low-fat dairy products. These findings provide insight into food preferences and dietary quality for a large group of veterans, which can inform future public health strategies aimed at improving the health and wellness of veterans across all racial and ethnic groups.

## Author contribution

The authors’ responsibilities were as follows – YL, X-MTN, FBH, LD, SBW, KI, DDW: designed the study; X-MTN, YL, SBW: wrote the manuscript; FBH, WCW, JMG, KC: were responsible for final content; and all authors: have read and approved the manuscript.

## Funding

This research is based on data from the VA Million Veteran Program supported by award MVP#000 from the Department of Veterans Affairs. This publication does not represent the views of the Department of Veterans Affairs or the United States government.

## Data availability

Data cannot be shared publicly because of VA policies regarding data privacy and security. Data contain potentially identifying and sensitive patient information. All relevant summary level data are included in the manuscript. For investigators with appropriate authorizations within the Department of Veterans Affairs, requests for data access can be made.

## Conflict of interest

The authors report no potential conflicts of interest.
