# Supplementary material for: Racial and Ethnic Disparities in Dietary Intake and Quality Among United States Veterans
Source: Curr Dev Nutr. 2024 Sep 29;8(10):104461. doi: 10.1016/j.cdnut.2024.104461 (PMC11530779; doi:10.1016/j.cdnut.2024.104461)
Supplement: Multimedia component 1 [file mmc1.docx]

**Racial and Ethnic Disparities in Dietary Intake and Quality Among U.S. Veterans – Supplemental Appendix**

**Xuan-Mai T. Nguyen**

**Table of Contents:**

Page 1: Table of Contents

Page 2: Supplemental Figure 1: Dietary Approaches to Stop Hypertension (DASH) Score for Asian Males and Females in the VA Million Veteran Program.

Page 3: Supplemental Table 1: Dietary Approaches to Stop Hypertension (DASH) score for Males and Females in the VA Million Veteran Program by Race and Ethnicity.

Page 4: Supplemental Table 2: Sensitivity Analyses for Dietary Approaches to Stop Hypertension (DASH) Score Among Males and Females in the VA Million Veteran Program by Missing Data Handling Methods.

Page 5: Supplemental Table 3: Dietary Intake for Males and Females in the VA Million Veteran Program by Race and Ethnicity.

Page 6: Supplemental Figure 2: Dietary Intake for Asian Males and Females in the VA Million Veteran Program (servings/day except sodium in grams/day).

Page 7-8: Full Acknowledgement List for the VA Million Veteran Program.

**Supplemental Figure 1. Dietary Approaches to Stop Hypertension (DASH) Score for Asian Males and Females in the VA Million Veteran Program**


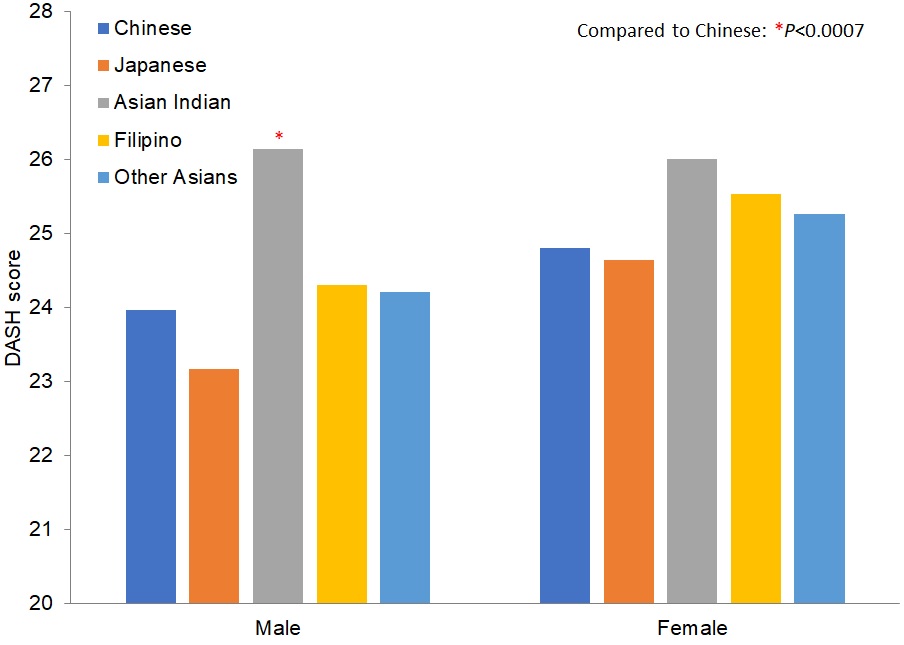


Age-adjusted means, ^*^*P*<0.0007 as compared to Asian Chinese after adjustment for age.

**Supplemental Table 1. Dietary Approaches to Stop Hypertension (DASH) score for Males and Females in the VA Million Veteran Program by Race and Ethnicity**

| **Race and ethnicity** | Non-Hispanic White | Non-Hispanic Black | Non-Hispanic Asian | Hispanic | Other |
| --- | --- | --- | --- | --- | --- |
| **MALES** |  |  |  |  |  |
| Age-standardized Mean  (SD) ^1^ | 23.75 (5.16) | 22.99 (5.21) | 24.37 (5.21) | 23.62 (5.20) | 23.47 (5.14) |
| *P* for age-adjusted differences | Reference. | <0.0001 | <0.0001 | 0.16 | <0.0001 |
| *P* for multi-adjusted differences^2^ | Reference. | <0.0001 | 0.73 | 0.30 | 0.82 |
| **FEMALES** |  |  |  |  |  |
| Age-standardized Mean  (SD) ^1^ | 25.33 (5.17) | 24.86 (5.32) | 25.71 (5.20) | 25.46 (5.28) | 25.17 (5.32) |
| *P* for age-adjusted differences | Reference. | <0.0001 | 0.82 | 0.06 | 0.44 |
| *P* for multi-adjusted differences^2^ | Reference. | 0.002 | 0.09 | 0.14 | 0.54 |

^1^Age-standardized mean (standard deviation (SD)) standardized to the age distribution of Non-Hispanic White participants.

^2^Multi-adjustment variates included age (<30, 30-39, 40-49, 50-59, 60-69, ≥70 (females), 70-79 and ≥80 (males), years), education level (≥some college: yes, no or missing), marital status (married or cohabitating with partner: yes, no or missing), annual household income (<$30,000, $30,000–$59,999, ≥$60,000, or missing), physical activity level (≥7.5 Mets-hours/week: yes/no), body mass index (<25, 25-29, , ≥30 kg/m^2^), and categories for branch of service (Army, Navy, Air Force, Marine Corps, and other). Significance level was set up at 0.0007 after Bonferroni correction for multiple comparisons.

**Supplemental Table 2. Sensitivity Analyses for Dietary Approaches to Stop Hypertension (DASH) Score Among Males and Females in the VA Million Veteran Program by Missing Data Handling Methods**^1^

| **Race and ethnicity** | Non-Hispanic White | Non-Hispanic Black | Non-Hispanic Asian | Hispanic | Other |
| --- | --- | --- | --- | --- | --- |
| **MALES** |  |  |  |  |  |
| Among all responders | 23.8 (5.4) | 22.5 (5.5)^*^ | 23.7 (5.6) | 23.3 (5.6)^*^ | 23.3 (5.5)^*^ |
| Among responders with no missing food items | 23.7 (5.2) | 23.0 (5.2)^*^ | 24.4 (5.3)^*^ | 23.6 (5.2) | 23.5 (5.2)^*^ |
| Among responders with no missing covariates | 24.0 (5.3) | 23.0 (5.4)^*^ | 24.1 (5.5) | 23.8 (5.42)^*^ | 23.9 (5.4)^*^ |
| **FEMALES** |  |  |  |  |  |
| Among all responders | 25.3 (5.3) | 24.8 (5.5)^*^ | 25.5 (5.4) | 25.4 (5.5) | 25.2 (5.4) |
| Among responders with no missing food items | 25.3 (5.2) | 24.9 (5.3)^*^ | 25.6 (5.1) | 25.5 (5.2) | 25.1 (5.4) |
| Among responders with no missing covariates | 25.5 (5.2) | 25.1 (5.4)^*^ | 26.5 (5.2) | 25.6 (5.4) | 25.6 (5.5) |

^1^Age-standardized mean (standard deviation (SD)) standardized to the age distribution of Non-Hispanic White participants ^*^*P*<0.0007 after Bonferroni correction for multiple comparisons as compared to Non-Hispanic White after adjustment for age (<30, 30-39, 40-49, 50-59, 60-69, ≥70 (females), 70-79 and ≥80 (males), years).

**Supplemental Table 3. Dietary Intake for Males and Females in the VA Million Veteran Program by Race and Ethnicity**

| **Race and Ethnicity** | Non-Hispanic White | Non-Hispanic Black | Non-Hispanic Asian | Hispanic | Other |
| --- | --- | --- | --- | --- | --- |
| **Dietary intake^*^** | Unadjusted | Age-standardized | Age-standardized | Age-standardized | Age-standardized |
| **MALES** |  |  |  |  |  |
| Total fruits, s/d | 1.45 (1.35) | 1.62 (1.72)^*^ | 1.70 (1.65)^*^ | 1.66 (1.68)^*^ | 1.52 (1.46)^*^ |
| Total vegetable, s/d | 1.29 (1.20) | 1.40 (1.63)^*^ | 1.58 (1.65)^*^ | 1.40 (1.57)^*^ | 1.37 (1.39)^*^ |
| Whole grains, s/d | 0.37 (0.56) | 0.38 (0.62) | 0.26 (0.42)^*^ | 0.31 (0.50)^*^ | 0.35 (0.56) |
| Nuts and beans, s/d | 0.83 (0.73) | 0.85 (0.84)^*^ | 0.81 (0.87)^*^ | 0.85 (0.83)^*^ | 0.85 (0.80)^*^ |
| Low-fat dairy, s/d | 0.81 (1.06) | 0.42 (0.82)^*^ | 0.48 (0.82)^*^ | 0.67 (1.05)^*^ | 0.68 (1.04)^*^ |
| Sodium, g/d | 1.38 (0.30) | 1.32 (0.29)^*^ | 1.18 (0.29)^*^ | 1.37 (0.31)^*^ | 1.36 (0.31)^*^ |
| Red and processed meat, s/d | 1.16 (0.88) | 1.21 (1.07)^*^ | 0.98 (0.85)^*^ | 1.11 (0.98)^*^ | 1.19 (0.97) |
| Sugar-sweetened beverages, s/d | 0.36 (0.78) | 0.44 (0.80)^*^ | 0.27 (0.61)^*^ | 0.33 (0.68)^*^ | 0.38 (0.80) |
| **FEMALES** |  |  |  |  |  |
| Total fruits, s/d | 1.45 (1.40) | 1.76 (1.87)^*^ | 1.73 (1.42) | 1.71 (1.76)^*^ | 1.60 (1.56)^*^ |
| Total vegetable, s/d | 1.53 (1.51) | 1.61 (1.71)^*^ | 1.81 (1.91) | 1.66 (1.71)^*^ | 1.62 (1.55) |
| Whole grains, s/d | 0.29 (0.46) | 0.27 (0.44) | 0.20 (0.31)^*^ | 0.26 (0.41) | 0.26 (0.42) |
| Nuts and beans, s/d | 0.85 (0.79) | 0.91 (0.90)^*^ | 0.88 (0.79) | 0.90 (0.86)^*^ | 0.92 (0.88)^*^ |
| Low-fat dairy, s/d | 0.83 (1.05) | 0.53 (0.81)^*^ | 0.61 (1.16)^*^ | 0.77 (1.10)^*^ | 0.68 (0.98)^*^ |
| Sodium, g/d | 1.22 (0.27) | 1.16 (0.26)^*^ | 1.11 (0.29)^*^ | 1.21 (0.28) | 1.20 (0.27) |
| Red and processed meat, s/d | 0.87 (0.74) | 0.90 (0.87) | 0.84 (0.76) | 0.82 (0.77)^*^ | 0.90 (0.81) |
| Sugar-sweetened beverages, s/d | 0.33 (0.82) | 0.42 (0.85)^*^ | 0.22 (0.48) | 0.30 (0.72) | 0.40 (0.95) |

Abbreviations: s/d: servings/day; g/d: grams/day; red meat includes both processed and unprocessed red meat.

^1^Unadjusted means (standard deviation) of Non-Hispanic White and age-standardized mean (standard error) of other race and ethnicity groups standardized to the age distribution of Non-Hispanic White participants.

^*^*P*<0.0007 after Bonferroni correction for multiple comparisons as compared to Non-Hispanic White after adjustment for age (<30, 30-39, 40-49, 50-59, 60-69, ≥70 (females), 70-79 and ≥80 (males), years), education level (≥some college: yes, no or missing), marital status (married or cohabitating with partner: yes, no or missing), annual household income (<$30,000, $30,000–$59,999, ≥$60,000, or missing), physical activity level (≥7.5 Mets-hours/week: yes/no), body mass index (<25, 25-29, ≥30 kg/m^2^), and categories for branch of service (Army, Navy, Air Force, Marine Corps, and others)

**Supplemental Figure 2. Dietary Intake of Asian Males and Females in the VA Million Veteran Program** (servings/day except sodium in grams/day)^1^ **
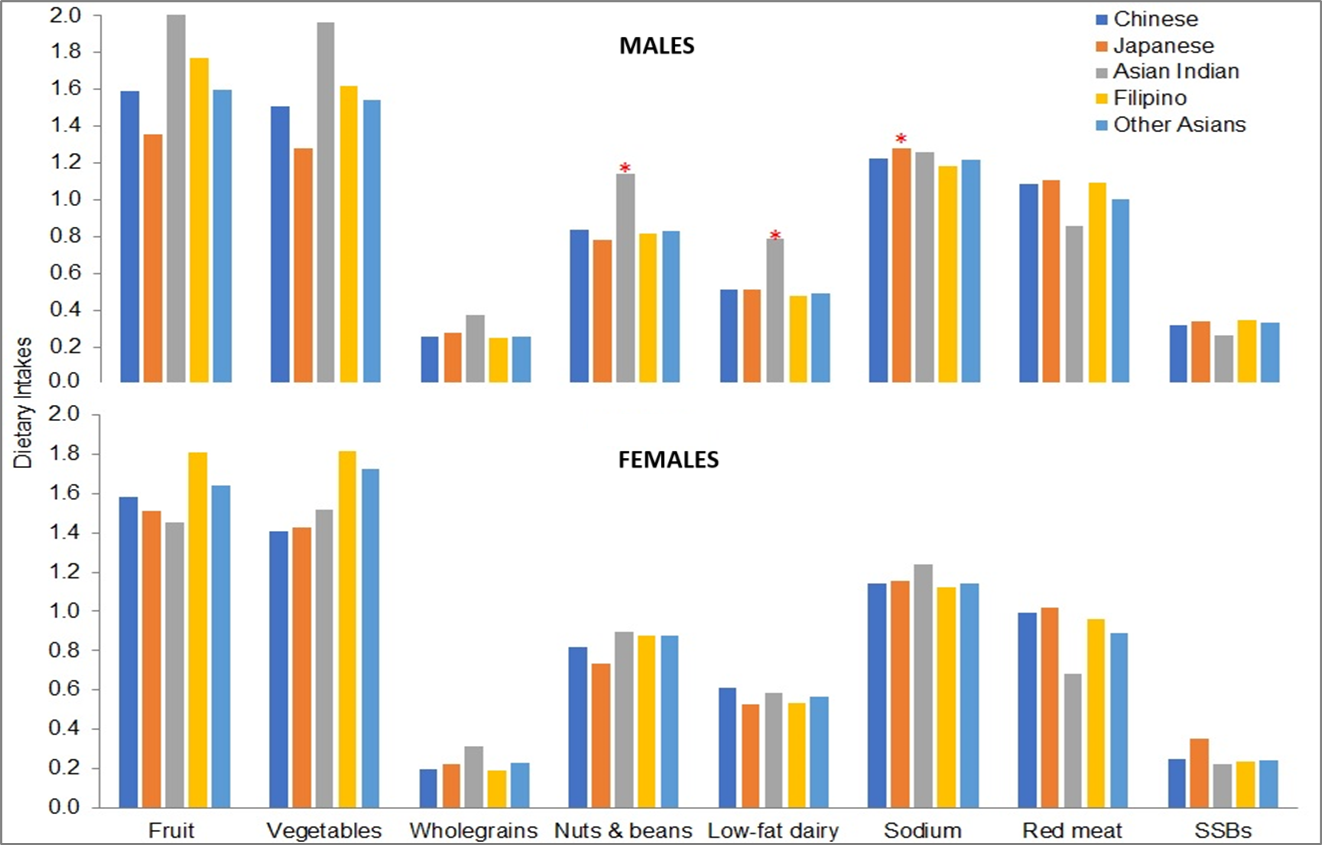
**

^1^age-adjusted means, ^*^*P*<0.0007 as compared to Chinese.

**VA Million Veteran Program:**

**MVP Program Office**

- Sumitra Muralidhar, Ph.D., Program Director

US Department of Veterans Affairs, 810 Vermont Avenue NW, Washington, DC 20420

- Jennifer Moser, Ph.D., Associate Director, Scientific Programs

US Department of Veterans Affairs, 810 Vermont Avenue NW, Washington, DC 20420

- Jennifer E. Deen, B.S., Associate Director, Cohort & Public Relations

US Department of Veterans Affairs, 810 Vermont Avenue NW, Washington, DC 20420

**MVP Executive Committee**

- Co-Chair: Philip S. Tsao, Ph.D.

VA Palo Alto Health Care System, 3801 Miranda Avenue, Palo Alto, CA 94304

- Co-Chair: Sumitra Muralidhar, Ph.D.

US Department of Veterans Affairs, 810 Vermont Avenue NW, Washington, DC 20420

- J. Michael Gaziano, M.D., M.P.H.

VA Boston Healthcare System, 150 S. Huntington Avenue, Boston, MA 02130

- Elizabeth Hauser, Ph.D.

Durham VA Medical Center, 508 Fulton Street, Durham, NC 27705

- Amy Kilbourne, Ph.D., M.P.H.

VA HSR&D, 2215 Fuller Road, Ann Arbor, MI 48105

- Michael Matheny, M.D., M.S., M.P.H.

VA Tennessee Valley Healthcare System, 1310 24^th^ Ave. South, Nashville, TN 37212

- Dave Oslin, M.D.

Philadelphia VA Medical Center, 3900 Woodland Avenue, Philadelphia, PA 19104

**MVP Co-Principal Investigators**

- J. Michael Gaziano, M.D., M.P.H.

VA Boston Healthcare System, 150 S. Huntington Avenue, Boston, MA 02130

- Philip S. Tsao, Ph.D.

VA Palo Alto Health Care System, 3801 Miranda Avenue, Palo Alto, CA 94304

**MVP Core Operations**

- Jessica V. Brewer, M.P.H., Director, MVP Cohort Operations

VA Boston Healthcare System, 150 S. Huntington Avenue, Boston, MA 02130

- Mary T. Brophy M.D., M.P.H., Director, VA Central Biorepository

VA Boston Healthcare System, 150 S. Huntington Avenue, Boston, MA 02130

- Kelly Cho, M.P.H, Ph.D., Director, MVP Phenomics

VA Boston Healthcare System, 150 S. Huntington Avenue, Boston, MA 02130

- Lori Churby, B.S., Director, MVP Regulatory Affairs

VA Palo Alto Health Care System, 3801 Miranda Avenue, Palo Alto, CA 94304

- Scott L. DuVall, Ph.D., Director, VA Informatics and Computing Infrastructure (VINCI)

VA Salt Lake City Health Care System, 500 Foothill Drive, Salt Lake City, UT 84148

- Saiju Pyarajan Ph.D., Director, Data and Computational Sciences

VA Boston Healthcare System, 150 S. Huntington Avenue, Boston, MA 02130

- Robert Ringer, Pharm.D., Director, VA Albuquerque Central Biorepository

New Mexico VA Health Care System, 1501 San Pedro Drive SE, Albuquerque, NM 87108

- Luis E. Selva, Ph.D., Director, MVP Biorepository Coordination

VA Boston Healthcare System, 150 S. Huntington Avenue, Boston, MA 02130

- Shahpoor (Alex) Shayan, M.S., Director, MVP PRE Informatics

VA Boston Healthcare System, 150 S. Huntington Avenue, Boston, MA 02130

- Brady Stephens, M.S., Principal Investigator, MVP Information Center

Canandaigua VA Medical Center, 400 Fort Hill Avenue, Canandaigua, NY 14424

- Stacey B. Whitbourne, Ph.D., Director, MVP Cohort Development and Management

VA Boston Healthcare System, 150 S. Huntington Avenue, Boston, MA 02130

**MVP Publications and Presentations Committee**

- Co-Chair: Themistocles L. Assimes, M.D., Ph. D

VA Palo Alto Health Care System, 3801 Miranda Avenue, Palo Alto, CA 94304

- Co-Chair: Adriana Hung, M.D.; M.P.H

VA Tennessee Valley Healthcare System, 1310 24^th^ Ave. South, Nashville, TN 37212

- Co-Chair: Henry Kranzler, M.D.

Philadelphia VA Medical Center, 3900 Woodland Avenue, Philadelphia, PA 19104
